# Evaluation of Flap Survival Using Local Glucose Measurement in Dogs Undergoing Reconstructive Procedures: Two Case Reports

**DOI:** 10.3390/vetsci13020143

**Published:** 2026-02-01

**Authors:** Daseul Kim, Sangyul Lee, Keuntae Lee, Kihoon Kim, Hwi-Yool Kim

**Affiliations:** 1 Department of Surgery, College of Veterinary Medicine, Konkuk University, Seoul 05029, Republic of Korea; 2 Department of Surgery, College of Veterinary Medicine, Kangwon National University, Chuncheon 24341, Republic of Korea

**Keywords:** reconstructive surgery, flap viability, flap perfusion, necrosis, dog

## Abstract

Early recognition of vascular compromise is essential for reconstructive flap survival. In human surgery, local glucose monitoring is widely used as an objective indicator of perfusion, but its application in veterinary patients is still limited. This report describes postoperative glucose measurement as a simple and minimally invasive method for evaluating flap viability in two dogs. This report describes two prospectively observed clinical cases in which local glucose measurement was applied as an adjunctive monitoring tool during postoperative flap management. Local glucose values were measured with a handheld glucometer at predefined flap and control sites. Serial readings were compared with daily assessments of flap color, temperature, turgor, and wound integrity. A previously suggested threshold of 60–62 mg/dL was used as a reference for potential perfusion compromise. In Case 1, a phalangeal fillet flap showed a brief glucose decline on postoperative days 2–3, followed by normalization and uneventful healing. In Case 2, which underwent advancement flap reconstruction after wound dehiscence, glucose values remained persistently below 60 mg/dL and preceded visible ischemia and distal necrosis. Local glucose monitoring provided rapid and clinically meaningful information about flap perfusion. Transient decreases reflected reversible postoperative congestion, whereas persistent hypoglycemia indicated progressive ischemia. These findings support the use of glucose monitoring as an adjunct in small-animal reconstructive surgery.

## Simple Summary

Successful healing of reconstructive skin flaps depends on maintaining adequate blood supply. In veterinary practice, evaluating flap viability often relies on subjective observations such as skin color or temperature, which may not detect early circulatory problems. In human reconstructive surgery, measuring local glucose levels has become an objective and minimally invasive method for identifying early signs of compromised blood flow. This report describes two dogs that underwent reconstructive surgery in which local glucose levels were measured postoperatively using a handheld glucometer. In the dog with normal healing, glucose values showed only a brief decline before returning to normal. In contrast, the dog that developed flap necrosis had persistently low glucose values throughout the monitoring period. These findings suggest that local glucose measurement may help veterinarians detect impaired flap circulation earlier than visual inspection alone, potentially improving treatment decisions and patient outcomes.

## 1. Introduction

In human reconstructive surgery, objective biochemical indicators such as local glucose concentration have been widely investigated as early markers of flap perfusion status. Previous human clinical studies have reported that glucose values near approximately 60–62 mg/dL are strongly associated with venous outflow compromise and impending flap failure [1]. Adequate vascular perfusion is fundamental to flap viability, and early detection of circulatory compromise is essential for successful reconstructive outcomes. Both diminished arterial inflow and impaired venous return can rapidly lead to irreversible ischemic injury if not recognized promptly.

In human medicine, declines in flap glucose levels have been shown to precede clinically observable signs of venous congestion or ischemia by several hours. Hara et al. identified glucose levels near approximately 62 mg/dL as strongly associated with venous outflow obstruction [1], while Henault et al. demonstrated that alterations in glucose and lactate reliably anticipate pedicle dysfunction [2].

Simplified indices such as the Glucose Index and ratio-based glucose change metrics have also been proposed as practical adjuncts for monitoring [3,4]. These concepts and cutoff values are derived exclusively from human reconstructive literature and are referenced here to provide physiological context rather than species-specific diagnostic criteria.

In reconstructive practice, intra- and postoperative glucose monitoring is attractive because handheld glucometers offer a rapid, inexpensive, and minimally invasive means of assessing metabolic status. Human studies have shown that glucose readings from point-of-care devices correlate closely with capillary or interstitial biochemical changes, enabling bedside assessment without specialized instrumentation [2,3]. This accessibility has facilitated broader adoption of glucose-based monitoring for assessing metabolic status in reconstructive practice.

Microdialysis investigations further reinforce the physiologic basis for glucose surveillance, demonstrating that glucose decline represents one of the earliest biochemical signatures of inadequate perfusion and precedes elevations in lactate, pyruvate, and other metabolites [5,6,7,8]. Continuous interstitial glucose sensors have recently expanded monitoring capability, enabling real-time assessment of flap metabolism before irreversible injury occurs [9,10].

Despite these advancements in human surgery [9,10], objective biochemical monitoring has not yet been widely adopted in veterinary reconstructive practice, which still relies predominantly on subjective assessment. Considering conserved microvascular physiology across species, integrating local glucose measurement into postoperative monitoring may enhance early detection of compromised flaps in canine patients. This report describes two canine cases utilizing local glucose monitoring to evaluate feasibility and clinical utility.

## 2. Materials and Methods

### 2.1. Animals and Case Selection

This study included two canine patients that underwent reconstructive surgery at the Veterinary Medical Teaching Hospital, Konkuk University. Both cases were prospectively observed, with local glucose monitoring applied as an adjunctive assessment during postoperative management following reconstructive procedures. Informed consent was obtained from all owners prior to treatment.

Case 1 was a 9-year-old, 8.1 kg, castrated male Poodle with a subcutaneous mass located on the right dorsal carpal region, cytologically diagnosed as a mast cell tumor. Preoperative evaluation included a complete blood count, serum biochemistry profile, thoracic imaging, abdominal ultrasonography, and computed tomography of the carpal region to assess local invasion and rule out metastatic disease.

Case 2 was a 10-year-old intact female Jindo dog weighing 20 kg with multiple mammary gland tumors involving the left second to fourth and right fourth to fifth mammary glands. Fine-needle aspiration suggested mammary neoplasia, and postoperative histopathology confirmed a grade 2 comedocarcinoma. Preoperative evaluation included a complete blood count, serum biochemistry profile, thoracic radiography, and abdominal ultrasonography, which documented concurrent findings including hepatic cysts and mild adrenal enlargement.

### 2.2. Anesthesia and Perioperative Management

Anesthetic protocols were individualized based on each patient’s physical status and anticipated surgical duration.

Case 1: Premedication consisted of midazolam (0.2 mg/kg IV; Bukwang Midazolam Inj., Bukwang Pharm., Seoul, Republic of Korea), fentanyl citrate (2 μg/kg IV; Fentanyl Citrate Inj. 10 mL, Hana Pharm., Seoul, Republic of Korea), and maropitant (1 mg/kg SC; Cerenia^®^, Zoetis, Parsippany, NJ, USA). Anesthesia was induced with propofol (6 mg/kg IV; Anepol Inj., Hana Pharm., Seoul, Republic of Korea) and maintained with isoflurane (Terrell™ Isoflurane, Kyongbo Pharm., Asan, Republic of Korea) in oxygen. Analgesia was achieved through a brachial plexus block using lidocaine (1% lidocaine; Daihan Lidocaine HCl Hydrate Inj. 1%, Daihan Pharm., Seoul, Republic of Korea) and a continuous fentanyl infusion (Fentanyl Citrate Inj. 10 mL, Hana Pharm., Seoul, Republic of Korea) during surgery. Perioperative antibiotic prophylaxis consisted of Cefazolin sodium (22 mg/kg IV; Chongkundang Cefazoline Inj. 1 g, Chongkundang Pharm., Seoul, Republic of Korea) administered according to standard dosing protocols. And meloxicam (0.2 mg/kg SC; Metacam^®^, Boehringer Ingelheim, Ingelheim, Germany) was provided for postoperative analgesia.

Case 2: Premedication and anesthetic protocol was performed in the manner as same as Case 1. Intraoperative analgesia was provided using a continuous infusion of fentanyl citrate (Fentanyl Citrate Inj. 10 mL, Hana Pharm., Seoul, Republic of Korea). Perioperative antibiotic therapy consisted of Amoxicillin–clavulanic acid (13.75 mg/kg IV; Amocla Inj., Kuhnil Pharm., Seoul, Republic of Korea) administered according to standard dosing protocols. Throughout anesthesia, the patient was monitored via non-invasive blood pressure, electrocardiography, respiratory rate, pulse oximetry, and body temperature measurements.

### 2.3. Surgical Procedure

**Figure 1:**
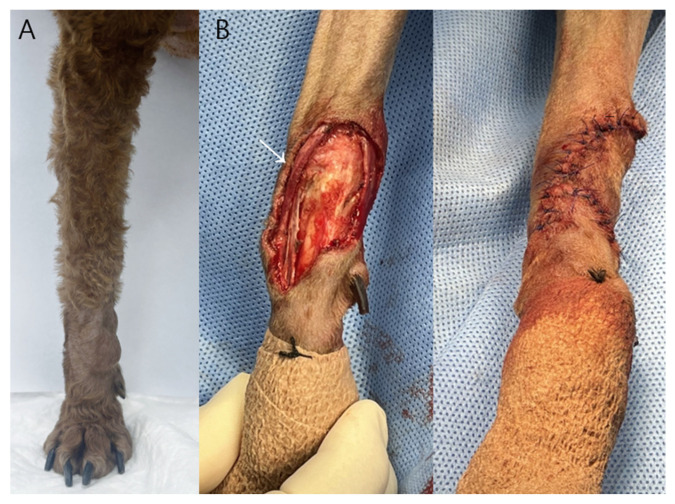
The surgical site of the right carpal joint mass in Case 1. (**A**) Preoperative photograph showing the subcutaneous mass on the right dorsal carpal region. (**B**) Intraoperative photograph demonstrating preservation of the right cephalic vein (white arrow) during tumor excision, with the mass isolated from the surrounding soft tissues. Figures were prepared using Microsoft PowerPoint (version 2019, Microsoft Corp., Redmond, WA, USA).

Case 1: A 2.0 × 1.4 × 1.0 cm ovoid mass located on the right dorsal carpal region was surgically excised with approximately 2 mm lateral margins due to anatomical constraints and the need to preserve the adjacent cephalic vein, rather than as an oncologically wide excision (Figure 1).

Following tumor removal, a phalangeal fillet flap was harvested from the first digit of the right forelimb to reconstruct the resultant skin defect. The flap dimensions provided adequate coverage without the need for additional tension-relieving techniques and were consistent with previously described techniques for reconstruction of carpal defects using phalangeal fillet tissue in dogs [11]. Subcutaneous tissues were apposed using a simple interrupted pattern with 3-0 polydioxanone suture (PDS II^®^, Ethicon, Somerville, NJ, USA), and the skin was closed using a simple interrupted pattern with 3-0 polyamide monofilament suture (Dafilon^®^, B. Braun, Melsungen, Germany). The region of greatest wound tension occurred over the carpal joint during flexion.

**Figure 2:**
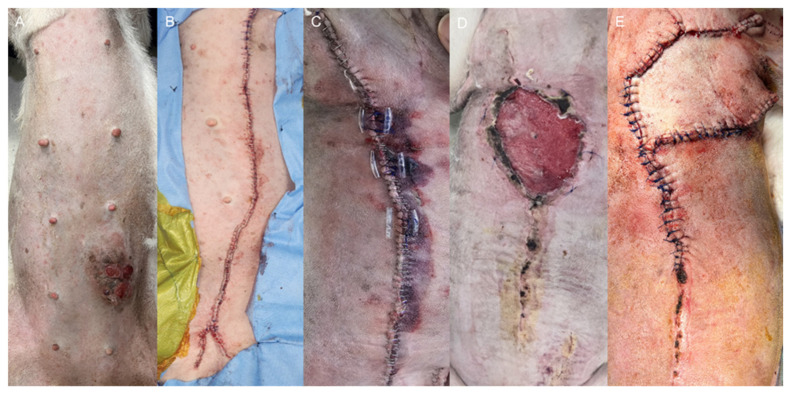
Surgical progression of mammary tumor resection and reconstruction in Case 2. (**A**) Preoperative mammary tumor site. (**B**) Left unilateral mastectomy and right fourth–fifth regional mastectomy. (**C**) Right first–third regional mastectomy performed five weeks later. (**D**) Wound dehiscence ten days after the second surgery. (**E**) Revision with a local advancement flap for tension-free closure.

Case 2: The patient underwent staged surgical management for multiple mammary gland tumors (Figure 2A), followed by reconstructive intervention. Initially, a left unilateral mastectomy and right 4th–5th regional mastectomy were performed (Figure 2B). Five weeks later, the second surgical stage was performed as a right 1st–3rd regional mastectomy, using the same operative technique with ligation of the associated superficial epigastric vessels (Figure 2C). Ten days after the second mastectomy, wound dehiscence developed due to excessive mechanical tension (Figure 2D). Revision surgery was performed using a local advancement skin flap to restore closure (Figure 2E). The flap was secured using a tension-relieving subcutaneous closure with 3-0 polydioxanone suture (PDS II^®^, Ethicon, Somerville, NJ, USA), followed by skin closure using a simple interrupted pattern with 3-0 polyamide monofilament suture (Dafilon^®^, B. Braun, Melsungen, Germany).

### 2.4. Local Glucose Measurement Protocol

Local glucose concentrations were measured in both cases using a handheld glucometer (FreeStyle Optium Neo, Abbott Diabetes Care, Alameda, CA, USA), with values recorded in mg/dL.

**Figure 3:**
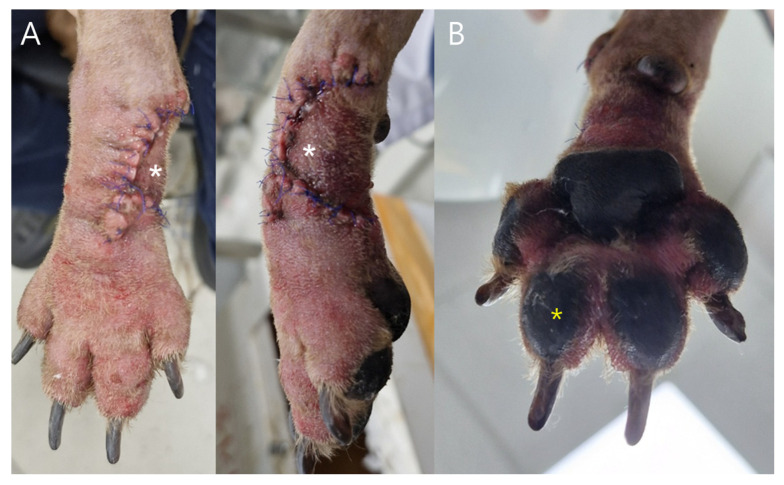
Phalangeal fillet flap site and glucose measurement sites in Case 1. (**A**) The white asterisk indicates the glucose sampling site located at the area of greatest mechanical tension within the phalangeal fillet flap (site 1). (**B**) The yellow asterisk indicates the ipsilateral third digital pad (site 2).

Case 1: Measurements were obtained from postoperative day (POD) 2 through POD 6 at three predefined sites: (1) the phalangeal fillet flap (white asterisk, Figure 3), (2) the ipsilateral third digital pad, (3) the contralateral third digital pad. Capillary blood samples were collected using a sterile lancet, and the first blood droplet was discarded to prevent dilution from interstitial fluid.

**Figure 4:**
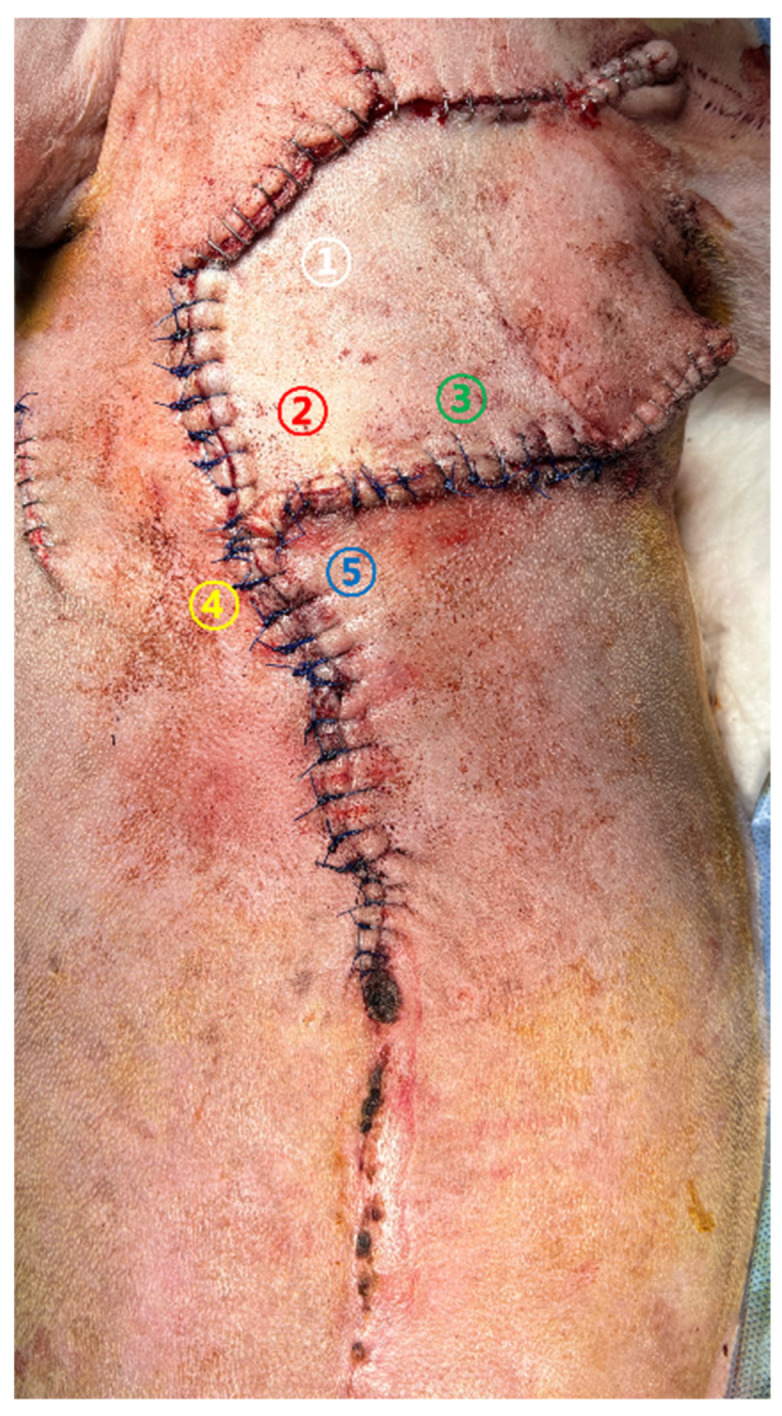
Five color-coded glucose measurement sites in Case 2. The white, red and green circles (sites ①–③) indicate central regions of the advancement flap, while the yellow and blue circles (sites ④, ⑤) represent peripheral areas near the wound margins.

Case 2. Glucose monitoring was performed at five color-coded sites (Figure 4) from the day of revision surgery to POD 5. Sampling was conducted following the same standardized protocol used in Case 1. Sites ①–③ represented central regions of the advancement flap, whereas sites ④ and ⑤ corresponded to peripheral areas adjacent to the wound margins.

Monitoring duration and measurement sites were determined on a case-by-case basis according to anatomical location, flap configuration, and clinical circumstances.

Measurement sites were selected to represent areas of maximal mechanical tension, central flap perfusion, and peripheral regions susceptible to ischemia. This approach was intended to capture spatial heterogeneity in flap perfusion and to facilitate interpretation of localized ischemic changes.

### 2.5. Postoperative Monitoring and Clinical Evaluation

Postoperative evaluations consisted of assessments of flap color, temperature, turgor, and capillary refill, with glucose measurements performed once daily. In Case 1, postoperative monitoring was conducted from postoperative day (POD)2 through POD7, whereas in Case 2, monitoring was performed from the day of revision surgery through POD21. Photographs were obtained once daily during the first postoperative week to document clinical progression.

**Figure 5:**
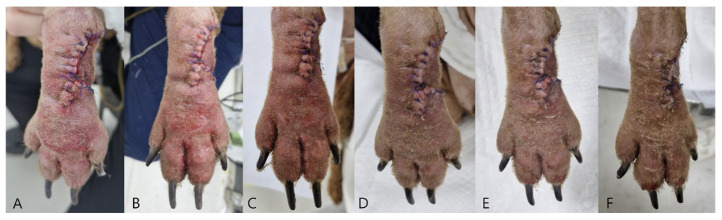
Serial postoperative photographs (POD2–7) of the phalangeal fillet flap over the right carpal region in Case 1. (**A**) POD2. (**B**) POD3. (**C**) POD4. (**D**) POD5. (**E**) POD6. (**F**) POD7. Mild edema and erythema evident on POD 2 gradually resolve over time.

Case 1: Postoperative clinical evaluation and glucose sampling were performed from POD 2 through POD7. Mild swelling and erythema gradually resolved by POD5–6 (Figure 5). Flap glucose levels showed an initial decrease during POD2–3 but subsequently stabilized, consistent with the uneventful healing and complete survival of the flap. Cefazolin sodium (22 mg/kg IV, three times daily; Chongkundang Cefazolin Inj. 1 g, Chongkundang Pharm., Seoul, Republic of Korea) was administered postoperatively for antimicrobial prophylaxis.

**Figure 6:**
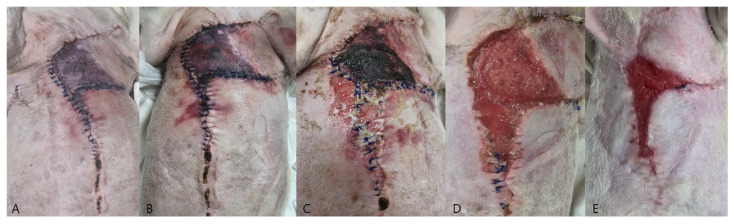
Postoperative photographs of the advancement flap from POD1 to POD21. (**A**) POD1. Early discoloration was first noted at the distal portion of the flap. (**B**) POD2. Areas of color discrepancy within the flap became clearly distinguishable. (**C**) POD5. Dehiscence developed at the distal portion of the flap. (**D**) POD10. Necrotic tissue was surgically debrided. (**E**) POD21. The compromised area ultimately healed by second intention with granulation tissue formation and epithelialization.

Case 2: Glucose values at multiple monitoring sites remained predominantly below 60 mg/dL throughout the postoperative period, with repeatedly recorded values below the detection threshold of the glucometer (<40 mg/dL) and measured nadir values as low as 3.8 mg/dL. These findings were accompanied by progressive distal flap necrosis and partial suture line dehiscence (Figure 6C). Healing of the compromised area ultimately occurred by second intention (Figure 6E). Amoxicillin–clavulanic acid (13.72 mg/kg IV, twice daily; Amocla Inj., Kuhnil Pharm., Seoul, Republic of Korea) was administered postoperatively for antimicrobial prophylaxis.

### 2.6. Ethical Considerations

This study involved client-owned canine patients that underwent standard diagnostic and therapeutic care, including reconstructive surgery, with additional local glucose monitoring performed as an adjunctive observational assessment. Local glucose measurement is not currently considered part of standard veterinary clinical practice but was applied prospectively with owner consent as an ancillary monitoring tool.

Because this work represents a descriptive prospective case report without experimental intervention or alteration of standard therapeutic management, institutional ethical review and approval were not required. Written informed consent was obtained from the owners prior to surgery for anesthesia, surgical procedures, and adjunctive postoperative monitoring, including local glucose measurement.

## 3. Results

### 3.1. Case 1

A 9-year-old castrated male Poodle underwent surgical removal of a right carpal mast cell tumor followed by reconstruction using a phalangeal fillet flap. Postoperative observation continued for seven days. The flap maintained normal viability throughout the monitoring period without signs of ischemia, infection, or wound complications.

#### 3.1.1. Local Glucose Trend

Glucose measurements obtained at the three monitoring sites in Case 1 are summarized in Table 1.

#### 3.1.2. Clinical Observation

Daily photographs from POD2 through POD7 (Figure 5) demonstrated progressive normalization of flap color and resolution of edema. No dehiscence, drainage, or local infection was noted. Complete epithelialization of the carpal defect was achieved by POD10. Histopathological examination of the excised mass in Case 1 confirmed a low-grade cutaneous mast cell tumor with complete surgical margins.

### 3.2. Case 2

A 10-year-old female Jindo dog underwent staged mastectomy procedures and later required reconstructive surgery using a local advancement flap following wound dehiscence. Glucose levels and flap appearance were monitored daily for five days after revision surgery.

#### 3.2.1. Local Glucose Trend

Serial glucose values measured at the flap site ①–⑤ are summarized in Table 2.

Immediately after surgery (OP), glucose values at most sites were markedly decreased (<60 mg/dL). Central sites (sites ①–③ consistently showed severely depressed readings from POD1 to POD4. Distal sites (sites ④, ⑤ demonstrated a further decline to 37 mg/dL on POD4, coinciding with the onset of suture failure and partial distal necrosis. Unlike Case 1, no subsequent rise in glucose values was observed, and low readings persisted throughout the monitoring period.

#### 3.2.2. Clinical Observation

Photographic documentation (Figure 6) revealed progressive ischemic deterioration beginning on POD1, including pallor, darkening, and necrosis of the wound margins.

By POD5, necrosis had expanded over the caudal aspect of the flap. These changes corresponded closely with persistently low glucose values at the same measurement sites. Histopathology of the mammary gland tumors revealed a grade 2 comedocarcinoma, consistent with the preoperative cytologic findings.

### 3.3. Comparative Summary

Evaluation of repeated glucose measurements closely paralleled the perfusion condition of the flaps in both dogs. Case 1 showed a transient and reversible glucose decline that paralleled benign postoperative inflammation, while Case 2 demonstrated persistently low values associated with progressive ischemic necrosis. In both dogs, flap glucose levels falling below approximately 60–62 mg/dL corresponded with compromised perfusion, consistent with thresholds reported in human clinical studies [1,2,3]. These findings collectively support the feasibility of local glucose monitoring as an early, objective indicator of flap viability in canine reconstructive procedures.

## 4. Discussion

The present report describes two canine cases in which local glucose monitoring was applied to evaluate postoperative flap perfusion. Glucose trends in both dogs strongly correlated with clinical progression, demonstrating the physiologic validity and clinical utility of this technique.

Extensive human literature has established local glucose concentration as a rapid and objective metabolic indicator of flap vascular status [1,2,3]. Glucose decline occurs earlier than lactate elevation, making it a leading biochemical marker of perfusion disturbance [2,3]. These findings are consistent with microdialysis investigations showing that flap ischemia produces early reductions in interstitial glucose and elevations in lactate and pyruvate, reliably signaling compromised perfusion before necrosis [5,6,7,8].

Case 1 demonstrated a transient glucose decrease <62 mg/dL during early postoperative inflammation, followed by spontaneous normalization consistent with reversible congestion. Case 2 showed persistently depressed values, matching patterns of sustained perfusion failure described in microdialysis studies [5,6,7,8].

Traditional clinical indicators including color, temperature, turgor or capillary refill are often insensitive to early perfusion disturbances [1,2]. Similar challenges in human reconstructive surgery have increased interest in objective biochemical monitoring. Handheld or bedside glucose measurement can detect perfusion compromise earlier than visual inspection. Early work showed that bedside glucose readings identify ischemia before overt discoloration or temperature change [1]. Subsequent studies confirmed that capillary glucose monitoring is highly sensitive for early venous congestion and often precedes other clinical indicators [12]. Clinical reports further demonstrated effective incorporation into routine postoperative surveillance [13]. More recent evidence verified that handheld glucometers detect perfusion abnormalities reliably without specialized equipment [14].

These advantages, including minimal invasiveness, rapid results, low cost, and suitability for repeated measurements, make handheld glucose assessment highly applicable in veterinary practice [1,3]. In both cases presented, these benefits enabled consistent monitoring.

One major clinical strength of glucose monitoring is early detection before irreversible tissue injury. In the present cases, interstitial glucose decline preceded visible clinical signs such as discoloration, delayed capillary refill, or necrosis [1,2,3,12,13,14,15]. This provides a critical window for corrective action. Early identification of perfusion compromise may allow timely implementation of adjunctive interventions, including flap warming, reduction of mechanical tension, suture release, local wound revision, pharmacologic vasodilation, anticoagulation, or prompt surgical exploration to restore vascular patency. Case 1 showed transient decreases that normalized with resolution of postoperative edema. Case 2 demonstrated persistently low values several days before macroscopic necrosis, emphasizing glucose monitoring as an early-warning indicator. In Case 2, wound dehiscence and excessive mechanical tension likely contributed to compromised perfusion and may represent an important differentiating factor between the two cases. However, persistently depressed glucose values preceded progressive tissue breakdown, suggesting that metabolic impairment reflected by glucose monitoring captured underlying perfusion failure beyond mechanical factors alone.

This study includes only two cases, limiting generalizability and preventing statistical threshold validation. Because glucose concentration reflects metabolic activity, measurements may be influenced by systemic factors such as hemodynamics, temperature, anesthetic effects, and stress. Intermittent capillary glucose assessment captures discrete snapshots rather than continuous physiologic trends [16]. Because glucose measurements were obtained once daily, temporal resolution was limited, and the exact onset of perfusion compromise could not be determined. Accordingly, the term “early detection” in this study refers to identification of metabolic changes preceding overt clinical deterioration rather than real-time or continuous surveillance. For both cases, glucose values < 62 mg/dL were interpreted as indicative of potential perfusion compromise, based on the cutoff established in a previous human study [1].

Importantly, the glucose cutoff value of approximately 60–62 mg/dL referenced in this study originates exclusively from human reconstructive literature and has not been validated as a diagnostic threshold in canine patients. Therefore, this value should be interpreted as a reference indicator to contextualize glucose trends rather than as a definitive cutoff for flap compromise in dogs.

Additional variability may arise from edema, fluctuating congestion, or heterogeneous microcirculation, and standardized monitoring protocols may help reduce such variability. Continuous interstitial glucose monitoring systems, which were not used in the present cases, represent a promising future direction for veterinary flap monitoring by providing real-time metabolic data with higher temporal resolution.

The physiologic parallels between canine and human flaps underscore the translational significance of glucose-based monitoring. Dogs provide a clinically relevant model due to similarities in vascular architecture and wound-healing dynamics. The present findings support that biochemical flap monitoring established in human reconstructive surgery can be effectively adapted to small-animal patients.

Across the two canine cases, local glucose measurement proved to be a practical and physiologically meaningful indicator of postoperative flap perfusion. Transient reductions followed by normalization corresponded to reversible congestion, whereas persistent depression reflected progressive ischemia and flap necrosis. These trends mirror patterns extensively documented in human reconstructive surgery [1] as well as microdialysis physiology [5]. Handheld glucose testing offers advantages including minimal invasiveness, low cost, broad accessibility, and rapid feedback. Although intermittent sampling presents limitations, continuous glucose monitoring technologies may further enhance diagnostic sensitivity and expand clinical applicability.

## 5. Conclusions

Local glucose monitoring provided a rapid, minimally invasive, and clinically informative assessment of flap perfusion in two canine reconstructive cases. Glucose trends closely reflected underlying vascular conditions: transient decreases with recovery versus persistent depression with ischemia. These findings align with human reconstructive literature, demonstrating glucose decline as an early indicator of perfusion compromise [1,2,3,12,13,14,15,16]. Given its practicality, glucose monitoring is a promising adjunct for veterinary postoperative flap surveillance. Larger controlled studies are needed to refine diagnostic thresholds, establish reference intervals, and evaluate continuous monitoring technologies.

## Figures and Tables

**Table 1:** Glucose measurements (mg/dL) from POD2 to POD6 at three monitoring sites in Case 1: (1) the phalangeal fillet flap, (2) the ipsilateral third digital pad, and (3) the contralateral third digital pad. Tables were prepared using Microsoft Word (version 2019, Microsoft Corp., Redmond, WA, USA).

Monitoring Sites	POD2	POD3	POD4	POD5	POD6
Right forelimb phalangeal fillet flap	133.2	61.2	61.2	48.6	63
Right 3rd digital pad	97.2	91.8	75.6	77.4	70.2
Left 3rd digital pad	-	97.2	64.8	79.2	77.4

**Table 2:** Glucose measurements (mg/dL) from immediately after surgery to POD5 at monitoring sites ① to ⑤ in Case 2. Values displayed as “Low” indicate glucose concentrations below the lower detection limit of the glucometer (<40 mg/dL).

Monitoring Sites	OP	POD1	POD2	POD3	POD4	POD5
①	Low	Low	Low	Low	Low	No blood
②	Low	Low	Low	36	Low	36
③	-	45	32.4	Low	Low	Low
④	75	71	68.4	91.8	73.8	-
⑤	42	25	57.6	64.8	3.8	-

## Data Availability

The original contributions presented in this study are included in the article. Further inquiries can be directed to the corresponding author.
